# *N*-alkyl-*N*-(phosphonoethyl) substituted (meth)acrylamides – new adhesive monomers for self-etching self-priming one part dental adhesive

**DOI:** 10.3762/bjoc.5.72

**Published:** 2009-12-02

**Authors:** Joachim E Klee, Uwe Lehmann

**Affiliations:** 1 Dentsply De Trey, De-Trey-Str. 1, 78467 Konstanz, Germany

**Keywords:** adhesion to enamel and dentin, hydrolytic stability, *N*-alkyl-*N*-(phosphonoethyl) substituted (meth)acrylamides, polymerization enthalpy, REM investigation

## Abstract

Novel *N*-alkyl-*N*-(phosphonoethyl) substituted mono-, bis- and tris(meth)acrylamides **3** were synthesized by two different three-step reactions and characterized by IR, ^1^H NMR and ^13^C NMR spectroscopy as well as refractive index and viscosity. The phosphonoethyl substituted (meth)acrylamide monomers show improved hydrolytic stability compared to carboxylic esters. The highest stability was found for the phosphonoethyl substituted acrylamide monomers. Acrylamides have a larger polymerization enthalpy ranging from −50 to −70 kJ·mol^−1^ per double bond compared to methacrylamides which show −8.57 to −25.1 kJ·mol^−1^ per double bond. Depending on their structure (meth)acrylamides **3** exhibit an adhesion to enamel and dentin up to 19.5 MPa. The monomer **3c** shows the highest adhesion values to both substrates, namely 15.3 ± 3.4 MPa to enamel and 18.5 ± 2.3 MPa to dentin.

## Introduction

In the past decades dental adhesives have been employed for fixation of direct and indirect restorations. These adhesives were composed of three-part systems, consisting of an etch gel, a primer and a bonding material. Each of these adhesive parts was applied step-by-step. At first the etch gel was applied to enamel and dentin surface, then the gel was removed by washing with water. Thereafter, the primer and the bonding were applied successively and light polymerized. This multi-step procedure is relatively time consuming and bears the danger of various failures. In order to reduce the complexity of the adhesives during application and to make the adhesion procedure more safe, easy and robust several generations of adhesives were developed which combined the etch and prime function or the prime and bond function in one part together.

At present, self-etching, self-priming dental adhesives are composed of two-part systems due to low hydrolytic stability of conventional polymerizable acidic ester monomers. The low hydrolytic stability arises from the hydrolysis of acidic and adhesive (meth)acrylester monomers in water or water/solvent mixtures. Therefore, the known acidic and adhesive monomers must be stored water-free and mixed with the aqueous part just before application. If conventional (meth)acrylesters are used for one-part self-etching, self-priming dental adhesives they necessitate a storage under refrigeration to guarantee a shelf-life, comparable to two-part systems. That is the case for the most of the presently available adhesives of this type. Today the demand is to have a one-part self-etching adhesive which combines all three steps of adhesive procedure in one and which does not need to be refrigerated. Therefore, there is a need for novel hydrolytically stable monomers with and without acidic functions.

It is well known that methacrylamides exhibit in acidic aqueous solutions an improved hydrolytic stability compared to ester containing monomers [1]. In the past different approaches were made for hydrolytically stable acidic monomers. Recently, some (*N*-methylacrylamido)alkylphosphonic acids [2], a 4-(*N*-methylacrylamidomethyl)benzylphosphonic acid [2] and a bis(meth)acrylamide comprising one phosphoric acid moiety [3] have been prepared and were investigated for a dental adhesive. Furthermore, (meth)acrylamides with one [4–8] or two [7–9] phosphonic acid groups were suggested as hydrolytically stable acidic monomers.

The aim of the presented work was to synthesize novel (meth)acrylamides with phosphonic acid moieties and to investigate the influence of monomers bearing different aliphatic side chains and a different number of polymerizable and acidic groups on the adhesion of enamel and dentin.

## Results and Discussion

**Scheme 1:**
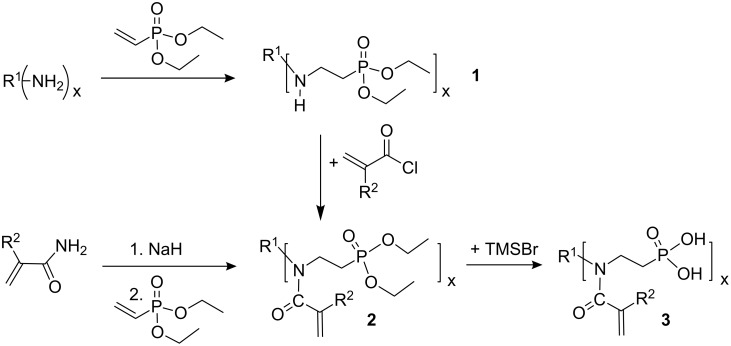
Synthesis of novel *N*-alkyl-*N*-(ethyl phosphonate) (meth)acrylamides **3**. For **2a**, **2b**, **3a**, **3b**: R^1^ = H, x = 1.

(Meth)acrylamides **3c**–**3f**, bis(meth)acrylamides **3g** and **3h** and tris(meth)acrylamides **3i** and **3j** were prepared in a three step reaction via addition of vinylphosphonic acid to amines, a Schotten-Baumann acylation with (meth)acrylolyl chloride and subsequent methanolysis of the phosphonic acid ethyl ester with trimethylsilyl bromide (Scheme 1, Table 1). (Meth)acrylamides **3a** and **3b** were prepared by addition of vinylphosphonic acid ethyl ester to acrylamide and methacrylamide respectively and the same procedure for methanolysis of the phosphonic acid ethyl ester as that described above.

**Table 1:** Formula, M_n_-values and yields of *N*-alkyl-*N*-(phosphonoethyl) (meth)acrylamides **3**.

**3**	R^1^	R^2^	x	formula	M_n_ g mol^−1^	Yield^c^ g (%)

**a**	H	H	1	C_5_H_10_O_4_NP	179.11	–
**b**	H	CH_3_	1	C_6_H_12_O_4_NP	193.14	2.0 (95.6)
**c**	C_4_H_9_	H	1	C_9_H_18_O_4_NP	235.22	32.2 (63.0)
**d**	C_4_H_9_	CH_3_	1	C_10_H_20_O_4_NP	249.25	36.8 (75.2)
**e**	C_8_H_17_	H	1	C_13_H_26_O_4_NP	291.33	33.3 (100.0)
**f**	C_12_H_25_	H	1	C_17_H_34_O_4_NP	347.44	25.1 (100.0)
**g**	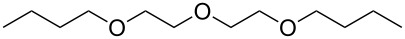	H	2	C_20_H_38_O_11_N_2_P_2_	544.48	21.0 (100.0)
**h**	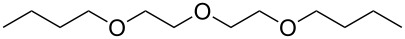	CH_3_	2	C_22_H_42_O_11_N_2_P_2_	572.53	16.9 (79.9)
**i**	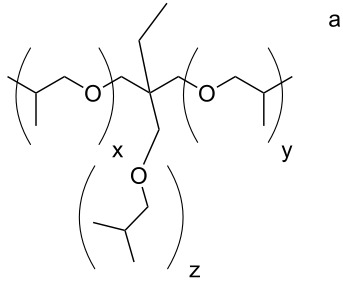	H	3	C_35_H_64_O_12_N_3_P	750.89	75.9 (100.0)
**j**	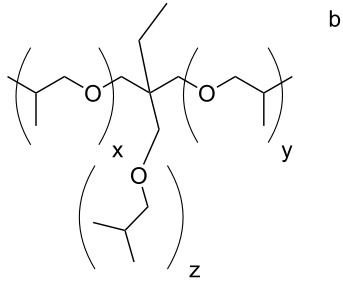	CH_3_	3	C_39_H_74_O_18_N_3_P_3_	968.96	27.1 (95.8)

^a^monophosphonic acid. ^b^triphosphonic acid. ^c^the yields refer to methanolysis of **2** to **3**.

The double bonds of **3** are detectable in the IR spectrum at 1533 cm^−1^ (**3f**) and in the ^1^H NMR spectrum at 5.3/5.5 ppm (**3d**). In the ^13^C NMR spectra signals of the sp^2^-hybridized C-atoms of the acrylamide double bonds appear at 128.9/129.1 (**3c**, **3e**, **3f**) and of the methacrylamides at 118.9/139.9 (**3b**), 115.7/141.2 ppm (**3d**), 121.7/140.2 ppm (**3h**). Absorptions of the P-OH moiety were found at 1078, 3390 and 3411 cm^−1^ (**3c**, **3d**).

### DSC investigation of **3**

The polymerization behavior of *N*-alkyl-*N*-(phosphonoethyl)acrylamides and methacrylamides **3** was investigated by photopolymerization using a DSC7/DPA7 unit. The polymerization enthalpy of acrylamides **3** (Table 2) is ranging from −50 to −70 kJ·mol^−1^ per double bond. It is only slightly lower compared to the polymerization enthalpy of acrylamide which was measured with −75.4 [10] and −82.9 kJ·mol^−1^ [11] and to acrylic esters (−77.5 to −80.5 kJ·mol^−1^ [12–13]). The longer the alkyl substituent and the higher the steric hindrance, the lower is the polymerization enthalpy of *N*-alkyl-*N*-(phosphonoethyl)acrylamides in the order of **3c**, **3e** and **3f**. The diacrylamide **3g** shows a relatively low polymerization enthalpy of −21.5 kJ·mol^−1^ per double bond when polymerized photochemically or thermally with AIBN as initiator.

**Table 2:** Polymerization enthalpy of *N*-alkyl-*N*-(phosphonoethyl) substituted (meth)acrylamides **3** using a photo calorimeter DSC7/DPA7 (Perkin-Elmer); light intensity in the visible portion of the spectrum was 108 mW cm^−2^.

**3**	**a**	**b**	**c**	**d**	**e**	**f**	**g**	**h**	**i**

Δ_R_*H* kJ·mol^−1^	−63.5	−8.6^a^	−71.3	−15.0^a^	−53.4	−51.1	−43.0^a^	−50.2	−179.8
Δ_R_*H* kJ·mol^−1^ per double bond	−63.5	−8.6	−71.3	−15.0	−53.4	−51.1	−21.5	−25.1	−59.9

^a^thermal polymerization initiated with AIBN.

The methacrylamides **3** exhibit a relatively strong delay of photopolymerization. The thermal initiated polymerization with AIBN leads to relatively low polymerization enthalpies ranging from −8.57 (**3b**) to −25.1 (**3h**) kJ·mol^−1^ per double bond only, which are significantly lower compared to methacrylic esters (−52.8 to −59.9 kJ·mol^−1^ [11–13]). Obviously, the steric hindrance of the methyl group and its electronic influence on the transition state limits the polymerization behavior. Already the polymerization enthalpy of methacrylamide is significantly lower (−35.2 kJ·mol^−1^ [12]) compared to methacrylic esters. Furthermore, it is well known, that *N*,*N*-disubstituted methacrylamides have relatively low reactivity in homopolymerization [14] due to the steric hindrance of the alkyl substituents.

### Hydrolytic stability of monomers **3**

It is well known that amides are more stable towards acidic hydrolysis than carboxylic ester compounds. On the other side it seems difficult to predict how stable the novel (meth)acrylamides are which contain, beside the (meth)acrylamide structures, acidic moieties within one molecule.

**Figure 1:**
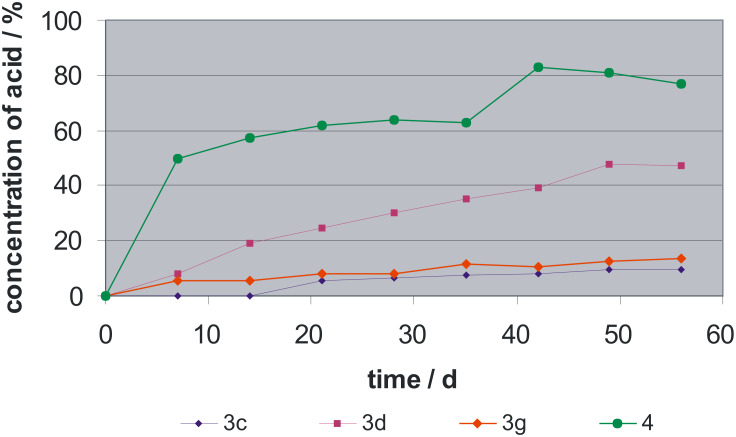
Hydrolysis of *N*-alkyl-*N*-(phosphonoethyl) substituted (meth)acrylamides **3** of Scheme 1 and of methacrylic acid phosphonomethyl ester (**4**) after storage in aqueous solution at 50 °C.

Hydrolytic stability was studied by detection of acrylic or methacrylic acid by HPLC using solutions of 1.5 mmol of **3c** and **3d** and solutions of 0.6 mmol **3g** in 5 mL of a 1:1 mixture of distilled water and ethanol which were stored at 50 °C (Figure 1). Methacrylic acid phosphonomethyl ester (**4**) shows the highest degree of hydrolysis of approximately 80% after 42 d at 50 °C. The lowest degree of hydrolysis was found for acrylic amide monomers **3c** and **3g**, whereas the methacrylic amide monomer **3d** exhibited a hydrolysis of 50% after 50 d at 50 °C. Obviously, the +I-effect of the methyl group in **3d** leads to a transition state which facilitates the amide cleavage.

### Adhesion of phosphonic acids **3**

**Figure 2:**
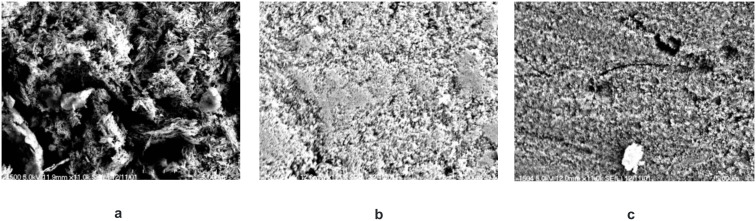
Etch pattern of enamel etched with a) Conditioner 36, magnification 11 × 10^3^, b) acidic monomer **3g** (*c* = 0.5 mol/l) in ethanol/water 1:1, magnification 11 × 10^3^, c) Clearfil SE Primer, magnification 11 × 10^3^.

One of the fundamental assumptions is that the adhesion of self-etching adhesives on enamel mainly depends on the acidity and consequently on the etch pattern that is formed. With the help of REM investigations the etch pattern of Conditioner 36, monomer **3g** and Clearfil SE Primer are compared in Figure 2a–Figure 2c which illustrates that it seems possible to generate a deeper and more structured etch pattern than the one caused by the Clearfil SE Primer. However, the strong etch pattern of Conditioner 36 is not surpassed.

**Figure 3:**
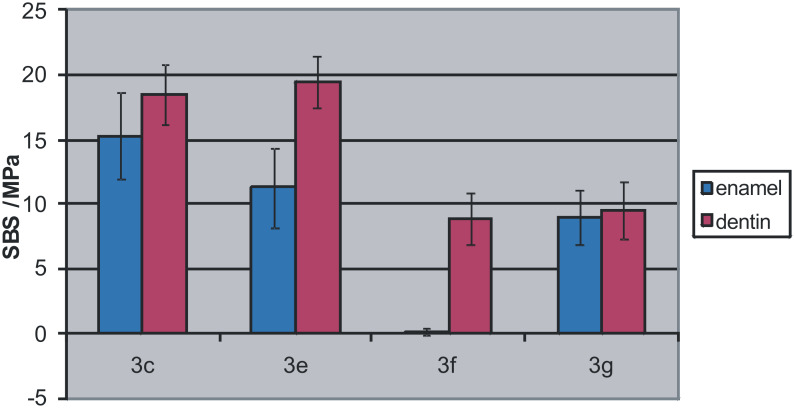
Shear bond strength (SBS) of phosphonic acids **3c**, **3e**, **3f** and **3g** in a formulation of an aqueous ethanol solution of *N*,*N*′-diethyl-1,3-bis(acrylamido)propane, phosphonic acid **3**, and 3(4),8(9)-bis(acrylamidomethyl)tricyclo[5.2.1.0^2,6^]decane.

The adhesion of phosphonic acids **3c**, **3e**, **3f** and **3g** was investigated using an aqueous ethanol solution of *N*,*N*′-diethyl-1,3-bis(acrylamido)propane, phosphonic acid **3**, and 3(4),8(9)-bis(acrylamidomethyl)tricyclo[5.2.1.0^2,6^]decane (Figure 3) which was applied to enamel and dentin, followed by solvent evaporation by (air stream) and photopolymerization. Surprisingly, phosphonic acid **3c** shows the highest adhesion to both enamel and dentin. Probably, **3c** fulfils better than the other monomers some of the essential conditions for high adhesion to both tooth substrates such as the presence of hydrophilic/hydrophobic moieties of the adhesive molecule and a high polymerization tendency.

Especially with increasing chain length of the alkyl substituent the adhesion to enamel decreases strongly. The dentin adhesion seems to be independent of the alkyl substituent in the range of four to eight carbon atoms. Only longer aliphatic side chains of twelve carbon atoms limit the adhesion of dentin. These results are surprising in the light of the work of Kuraray [15] which showed a maximum of adhesion for relatively long C-10 alkyl spacers.

In order to further improve the adhesion, polyfunctional monomers **3g**–**j** were synthesized. It was expected that the adhesion of **3g**, which contains two polymerizable moieties and two phosphonic acid groups, is superior compared to the monofunctional monomers. However, the adhesion of **3g** is balanced on both enamel and dentin, but amounts to less than 10 MPa on each substrate only. Maybe the rather poor polymerization behavior and the relatively high molecular weight are responsible for the lower adhesion.

## Conclusion

A series of novel *N*-alkyl-*N*-(phosphonoethyl) substituted mono-, bis- and tris(meth)acrylamides **3** was synthesized which shows improved hydrolytic stability compared to carboxylic esters. Their stability decreases in the order of acrylamide **3c** ~ **3g** > methacrylamide **3d** > methacryl ester **4**. Acrylamides show a higher polymerization enthalpy ranging from −50 to −70 kJ·mol^−1^ per double bond compared to methacrylamides which show only −8.57 to −25.1 kJ·mol^−1^ per double bond. The (meth)acrylamides **3** exhibit an adhesion to both enamel and dentin. The highest adhesion values to both tooth substrates were found using **3c** with 15.3 ± 3.4 MPa to enamel and with 18.5 ± 2.3 MPa to dentin.

## Experimental

Diethyl vinylphosphonate, acryloyl chloride, methacryloyl chloride, methacrylic acid, methylene chloride, NaOH, trimethylbromosilane, butylamine, 3,6-dioxaoctane-1,8-diamine, camphorquinone and 4-(*N*,*N*-dimethylamino)benzoic acid ethyl ester, diethyl (hydroxymethyl)phosphonate, 2,2′-azobisisobutyronitrile (AIBN) and 2,6-di-*tert*-butyl-4-methylphenol were purchased from Sigma-Aldrich and phenylbis(2,4,6-trimethylbenzoyl)phosphine oxide from BASF Interorgana. *N*,*N*′-diethyl-1,3-(bisacrylamido)propane, 3(4),8(9)-bis(acrylamidomethyl)tricyclo[5.2.1.0^2,6^]decane all of Dentsply. Spectrum TPH (Dentsply) is a dental composite comprising methacrylate basing resins and glass filler. Nupro (Dentsply) is a polishing paste. Conditioner 36 (Dentsply) mainly consist of phosphoric acid. Clearfil SE (Kuraray) is a water-free two-part self-etching adhesive containing 10-(methacryloyloxy)decyl dihydrogen phosphate.

The two (meth)acrylamido phosphonic acids **3a** and **3b** (Table 1) were synthesized according to the following general procedure.

**1. (Meth)acrylamido phosphonic acid diethyl ester 2a and 2b:** To a solution of acrylamide or methacrylamide in 30 mL CH_2_Cl_2_ were added a stoichiometric amount of NaH at 0 °C. After 1 h a stoichiometric amount of vinyl phosphonic acid diethyl ester was added to the reaction mixture.

**2. (Meth)acrylamido phosphonic acids 3a and 3b:** To the (meth)acrylamidoethylphosphonic acid diethyl ester dissolved in 10 mL methylene chloride was added trimethylbromosilane over a period of 20 min under stirring. Thereafter the reaction mixture was stirred for additional 3 h. The phosphonic acid silyl esters were hydrolyzed by adding methanol, extracted with CH_2_Cl_2_ and dried over MgSO_4_. Prior to removing the solvent 0.025 mol % 2,6-di-*tert*-butyl-4-methylphenol was added. The product was dried at 40 °C in vacuum.

The four monoalkyl (meth)acrylamido phosphonic acids **3c**–**f**, the bis(meth)acrylamido phosphonic acids **3g**–**h** and the tris (meth)acrylamido phosphonic acids **3i**–**j** (Table 1) were synthesized according to the following general procedure.

**1. Diethyl *N*-alkyl-2-aminoethylphosphonate:** Diethyl vinylphosphonate was added to the alkyl amine and refluxed for 24 h at 65 °C. After this time signals of the vinyl group at 134.9 (CH_2_=) and 127.3/124.3 ppm (CH=) are completely missing from the ^13^C NMR spectrum.

**2. *N*-Alkyl-*N*-[2-(diethylphosphono)ethyl]-(meth)acrylamide:** In a 1 litre – 4-necked flask equipped with stirrer, thermometer and two 50 mL dropping funnels *N*-alkyl-2-aminoethylphosphonate was dissolved in 100 mL methylene chloride and cooled down to 0–5 °C. Acryloyl chloride or methacryloyl chloride dissolved in 130 mL methylene chloride and NaOH dissolved in 200 mL water was dropped simultaneously under stirring to this solution so that the temperature did not rise above 5 °C. Thereafter, the mixture was stirred for additional 2 h at room temperature. The reaction mixture was extracted twice with 150 mL 1 M HCl and 150 mL 1 M NaHCO_3_. Then the organic phase was washed with 150 mL deionized water, until the water had a pH-value of approximately 7. The separated organic phase was dried with Na_2_SO_4_ over night. The Na_2_SO_4_ was filtered off and extracted with methylene chloride. After addition of 0.025 mol % 2,6-di-*tert*-butyl-4-methylphenol relative to the (meth)acrylamide the methylene chloride was distilled off using a rotation evaporator until 8 mbar.

**3. *N*-Alkyl-*N*-(2-phosphonoethyl)-(meth)acrylamide:** In a 4-necked 1 litre flask equipped with a stirrer, a thermometer, refluxer with CaCl_2_-drying tube and 50 mL dropping funnels *N*-alkyl-*N*-[2-(diethylphosphono)ethyl]-(meth)acrylamide was dissolved in 100 mL of methylene chloride. Then trimethylbromosilane was added dropwise over a period of 20 min under stirring. Thereafter the reaction mixture was stirred for additional 2 h. By adding of 100 mL methanol the phosphonic acid silyl esters were hydrolyzed. Prior to removing the solvents 2,6-di-*tert*-butyl-4-methylphenol was added. The product was dried at 40 °C in vacuum.

**2-(Acrylamido)ethylphosphonic acid (3a)** was synthesized as described in [16].

**2-(Methacrylamidoethyl)phosphonic acid (3b):**


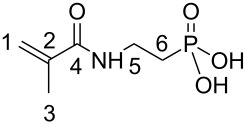


^13^C NMR (CH_3_OH-*d*4): δ = 166.5 (4), 139.9 (2), 118.9 (1), 34.7 (6), 28.2/26.9 (5), 18.8 (3) ppm.

***N*-Butyl-*N*-(2-phosphonoethyl)acrylamide (3c):**


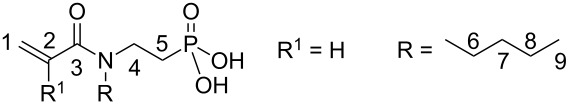


Soluble in CHCl_3_, CH_3_OH, C_2_H_5_OH, CHCl_3_/CH_3_OH, CH_3_OH/H_2_O, C_2_H_5_OH/H_2_O, acetone/water (1:1) and insoluble in acetone. mp = 129.7 °C. IR: 3411, 3390 (OH), 2973, 2929, 2885 (CH_2_/CH_3_), 1390 (CH_2_/CH_3_), 1078 cm^−1^ (OH). ^1^H NMR (CH_3_OH-*d*4): δ = 5.8 (P-OH), 5.7 (2), 5.3 (1), 3.9 (6), 4.45 (7), 2.7–2.3 (4, 5), 1.94 (8), 1.6 (9) ppm. ^13^C NMR (CH_3_OH-*d*4): δ = 168.4 (3), 129.1 (2), 128.9 (1), 43.8 (6), 32.6 (7), 30.8 (4), 27.9/25.8 (5), 21.2 (8), 14.2 (9) ppm. ^31^P NMR (CH_3_OH-*d*4): δ = 24.3/25.8 ppm.

***N*-Butyl-*N*-(2-phosphonoethyl)methacrylamide (3d):**


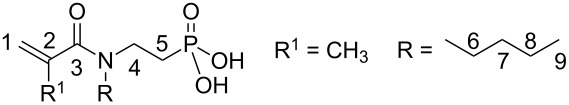


Soluble in CHCl_3_, CH_3_OH, C_2_H_5_OH, CHCl_3_/CH_3_OH, CH_3_OH/H_2_O and C_2_H_5_OH/H_2_O. IR: 3411, 3390 (OH), 2973, 2929, 2885 (CH_2_/CH_3_), 1390 (CH_2_/CH_3_), 1078 cm^−1^ (OH). ^1^H NMR (CH_3_OH-*d*4): δ = 5.85 (P–OH), 5.5/5.3 (1), 3.9 (6), 3.65 (7), 2.2–2.4 (5), 1.84 (8), 1.6 (9), 1.2 (R^1^ = CH_3_) ppm. ^13^C NMR (CH_3_OH-*d*4): δ = 174.5 (3), 141.7 (2), 115.7 (1), 44.1 (6), 31.6 (4), 30.5 (7), 27.0/25.1 (5), 20.6 (8), 20.3 (R^1^=CH_3_), 13.8 (9) ppm.

***N*-Octyl-*N*-(2-phosphonoethyl)acrylamide (3e):**


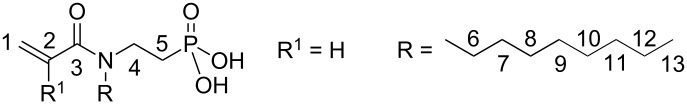


Soluble in CHCl_3_, CH_3_OH, C_2_H_5_OH, CHCl_3_/CH_3_OH, CH_3_OH/H_2_O, and C_2_H_5_OH/H_2_O. mp = 100.9 °C. IR: 3411, 3390 (OH), 2973, 2929, 2885 (CH_2_/CH_3_), 1390 (CH_2_/CH_3_), 1078 cm^−1^ (OH). ^1^H NMR (CH_3_OH-*d*4): δ = 6.6 (P–OH), 6.1 (2), 5.6 (1), 4.9 (6), 3.65 (7), 3.2–3.5 (4, 5, 8–11), 1.95 (12), 1.5 (13) ppm. ^13^C NMR (CDCl_3_): δ = 168.4 (3), 129.1 (2), 128.9 (1), 43.8 (6), 33.1 (7), 30.7 (4), 32.5 (11), 30.3 (9, 10), 28.7 /28.0 (8), 27.9/27.5 (5), 23.7 (12), 17.0 (13) ppm.

***N*-Dodecyl-*N*-(2-phosphonoethyl)acrylamide (3f):**


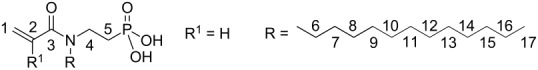


Soluble in CHCl_3_, CH_3_OH, C_2_H_5_OH, CHCl_3_/CH_3_OH, CH_3_OH/H_2_O, C_2_H_5_OH/H_2_O (50/50) and insoluble in H_2_O. mp = 89.7 °C. IR: 2919, 2853 (CH_2_/CH_3_), 1641 (CO), 1533 (C=C), 1469, 1443, 1377 (CH_2_/CH_3_), 1183 (P–OH), 794 (C=C) cm^−1^. ^1^H NMR (CH_3_OH-*d*4): δ = 6.4 (P–OH), 6.1 (2), 5.6 (1), 4.8 (6), 3.7 (7), 3.3–3.6 (4, 5, 8–15), 1.9 (16), 1.5 (17) ppm. ^13^C NMR (CDCl_3_): δ = 168.4 (3), 129.1 (2), 128.9 (1), 43.8 (6), 33.1 (7), 32.5 (15), 30.7 (4), 30.3 (9–14), 28.7 /28.0 (8), 27.9/27.5 (5), 23.7 (16), 17.0 (17) ppm.

***N*,*N*′-Bis(2-phosphonoethyl)-*N*,*N*′-diacryloyl-4,7,10-trioxatridecane-1,13-diamine (3g):**


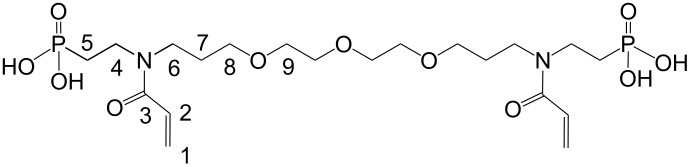


Soluble in CHCl_3_, CHCl_3_/CH_3_OH, CH_3_OH, C_2_H_5_OH, CH_3_OH/H_2_O and C_2_H_5_OH/H_2_O (50/50).


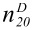
 = 1.5157 ± 0.0002, η_23 °C_ = 436 ± 7 Pa·s

IR: 3373 (POH), 2933, 2877 (CH_2_/CH_3_), 1714, 1638 (CO), 1586 (C=C), 1460, 1437, 1370 (CH_2_/CH_3_), 1183 (P–OH), 794 (C=C) cm^−1^. ^1^H NMR (CH_3_OH-*d*4): δ = 7.4 (P–OH), 5.2 (2), 5.1 (1), 4.5 (8, 9), 3.6 (6), 3.2–3.3 (4, 5, 7) ppm. ^13^C NMR (CDCl_3_): δ = 164.7 (3), 140.2 (2), 121.7 (1), 70.6 (9), 69.4 (8), 48.2 (6), 32.5 (4) 31.9 (7), 29.5 (5) ppm.

***N*,*N*′-Bis(2-phosphonoethyl)-*N*,*N*′-bis(methacryloyl)-4,7,10-trioxatridecane-1,13-diamine (3h):**


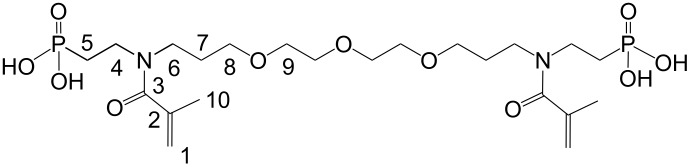


Soluble in CHCl_3_, CHCl_3_/CH_3_OH, CH_3_OH, C_2_H_5_OH, CH_3_OH/H_2_O and C_2_H_5_OH/H_2_O (50/50).


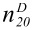
 = 1.4900, η_23 °C_ = 14.7 ± 0.3 Pa·s

IR: 3365 (POH), 2920, 2863 (CH_2_/CH_3_), 1722 (CO), 1584 (C=C), 1480, 1437, 1372 (CH_2_/CH_3_), 1109 (P–OH), 794 (C=C) cm^−1^. ^1^H NMR (CH_3_OH-*d*4): δ = 7.4 (P–OH), 5.1/5.2 (1), 4.5 (8, 9), 3.8 (6), 3.4–3.7 (4, 5, 7), 1.96 (10) ppm. ^13^C NMR (CDCl_3_): δ = 164.7 (3), 140.2 (2), 121.7 (1), 70.6 (9), 69.4 (8), 48.2 (6), 32.5 (4) 31.9 (7), 29.5 (5), 19.3 (10) ppm.

***N*-(2-Phosphonoethyl)-*N*,*N*′,*N*″-triacryloyl Jeffamine T403 (3i):**

Soluble in CHCl_3_, CHCl_3_/CH_3_OH, CH_3_OH, C_2_H_5_OH, CH_3_OH/H_2_O and C_2_H_5_OH/H_2_O (50/50) and insoluble in acetone. The pH value of a solution in C_2_H_5_OH/H_2_O =50/50 is 1.75.


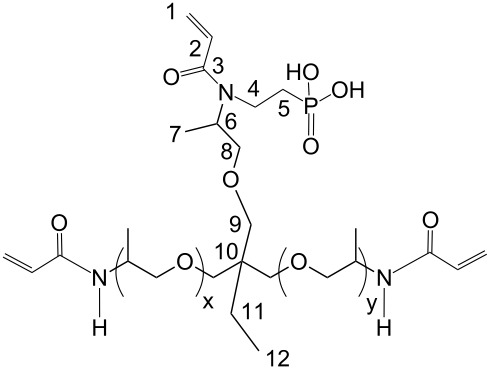



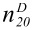
 = 1.4924 ± 0.0003, η_23 °C_ = 0.517 ± 0.018 Pa·s

IR: 3269 (POH), 2972/2933/2877 (CH_2_/CH_3_), 1734 (CO), 1652 (C=C), 795 (C=C), cm^−1^. ^1^H NMR (CH_3_OH-*d*4): δ = 6.8 (P–OH), 6.2/6.3 (2) 5.6/5.7 (1), 4.9 (8), 4.2 (9), 3.5–3.7 (4–6, 11), 1.4 (7), 1.2 (12) ppm. ^13^C NMR (CH_3_OH-*d*4): δ = 167.5 (3), 132.3 (2), 126.6 (1), 76.6 (8), 76.3 (9), 72.7 (6), 46.3 (5), 44.6 (4), 40.0 (10), 24.0 (11), 17.7 (7), 8.1 (12) ppm. ^31^P NMR (CH_3_OH-*d*4): δ = 29.5/29.9 ppm.

***N*,*N*′,*N*″-Tris(phosphonoethyl)-*N*,*N*′,*N*″-trismethacryloyl Jeffamine T403 (3j):**


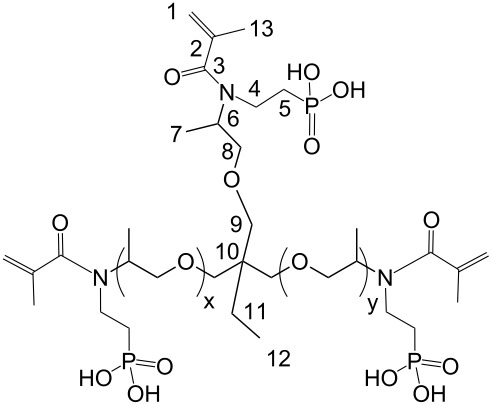


Soluble in CHCl_3_, CHCl_3_/CH_3_OH, CH_3_OH, C_2_H_5_OH, CH_3_OH/H_2_O and C_2_H_5_OH/H_2_O (50/50) and insoluble in acetone. The pH value of a solution in C_2_H_5_OH/H_2_O =50/50 is 1.75.


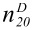
 = 1.4927 ± 0.0004, η_23 °C_ = 823 ± 1 Pa·s

IR: 3269 (POH), 2972/2933/2877 (CH_2_/CH_3_), 1734 (CO), 1652 (C=C), 795 (C=C), cm^−1^. ^1^H NMR (CH_3_OH-*d*4): δ = 6.1 (P–OH), 5.6 (1), 5.1 (8), 4.2 (9), 3.3–3.6 (4–6, 11), 1.9 (13), 1.4 (7), 1.1 (12) ppm. ^13^C NMR (CDCl_3_): δ = 174.9 (3), 140.7 (2), 115.7 (1), 121.7 (11), 70.6 (7), 69.4 (6), 62.3 (2), 48.2 (5), 32.5 (4), 25.1 (3), 19.3 (10), 16.4 (1) ppm. ^31^P NMR (CH_3_OH-*d*4): δ = 21.0/21.3 and 24.6/26.0 ppm.

**Methacrylic acid phosphonomethyl ester (4):**


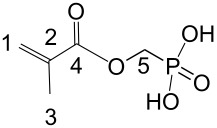


This compound was synthesized by esterification of methacrylic acid with diethyl (hydroxymethyl)phosphonate and subsequent methanolysis of the phosphonic acid diethylester with trimethylbromosilane according to the procedure described for monomer **3b**. Yield: 12.5 g (98.5% of th.), Δ_R_*H* = −39.0 kJ·mol^−1^. ^13^C NMR: δ = 166.8/166.6 (4), 134.3 (2), 127.4/125.2 (1), 58.7/56.0 (5), 17.6/17.4 (3) ppm.

The IR spectra were measured by using a FT-IR spectrometer (Nicolet 6700 FT-IR spectrometer (Thermo Scientific). The ^1^H NMR, ^13^C NMR and ^31^P NMR spectra were obtained by employing Bruker AC 250 MHz equipment. The viscosities were measured with the help of a Bohlin-Rheometer CS-50 at 23 °C.

The melting temperatures were measured by using a DSC 7 (Perkin-Elmer). The polymerization enthalpy was conducted in the isothermal mode at 37 °C, using the photo calorimeter DSC7/DPA7 (Perkin-Elmer). The light intensity in the visible portion of the spectrum was 108 mW·cm^−2^. Each DSC experiment included a short dark period (typically 6 s) and the subsequent illumination period. After the first run, an additional run was started using the polymerized material under the same experimental conditions. The subtraction of these runs from one another removed the effect of different baselines for the dark and the illumination periods. In the monomers were dissolved 0.3 mol % camphorquinone and 0.35 mol % 4-(*N*,*N*-dimethylamino)benzoic acid ethyl ester.

The hydrolytic stability was studied by detection of acrylic or methacrylic acid in HPLC using solutions of 1.5 mmol of **3c**, **3d** and **3e** and solutions of 0.6 mmol **3g** in 5 mL of a 1:1 mixture of distilled water and ethanol which were stored at 50 °C.

The adhesion on enamel and dentin of phosphonic acids **3** was tested using the following formulation: 0.2499 g *N*,*N*′-diethyl-1,3-bis(acrylamido)propane, 0.3000 g phosphonic acid **3**, 0.4168 g 3(4),8(9)-bis(acrylamidomethyl)tricyclo[5.2.1.0^2,6^]decane, 0.0071 g camphorquinone, 0.0083 g 4-(*N*,*N*-dimethylamino)benzoic acid ethyl ester and 0.0179 g bis bis(2,4,6-trimethylbenzoyl)-phenylphosphine oxide were dissolved in a solvent mixture composed of 0.5000 g ethanol and 0.5000 g water.

The following procedure was applied prior to adhesion measurement:

- At first teeth were abraded by 200 and 500 grit abrasive paper.
- Then the teeth were stored at 37 °C in water.
- Thereafter, teeth were treated with adhesive formulation for 20 s and the solvents were evaporated by air stream for 10 s.
- Now a light curing of adhesive layer occurred for 20 s.
- A polymerized Spectrum TPH body applied on the adhesive was cured on tooth three times for 20 s.
- At last the prepared teeth were stored in water at 37 °C for 2 h before measured.

REM investigation was done according to the following procedure: Tooth samples were polished with Nupro (Dentsply), etched with Conditioner 36 (Dentsply) or ethanol/water (1:1) solution of **3g** (*c* = 0.5 mol l^−1^), for 30 s, washed with water for 10 s and with ethanol for another 10 s. Then the teeth were dried in an exsiccator under vacuum for 4 d.
